# Supplementary material for: Complexome profiling of the Chlamydomonas psb28 mutant reveals TEF5 as an early PSII assembly factor
Source: Plant Cell. 2025 Mar 17;37(6):koaf055. doi: 10.1093/plcell/koaf055 (PMC12164590; doi:10.1093/plcell/koaf055)
Supplement: koaf055_Supplementary_Data [file koaf055_supplementary_data.zip › psb28_interactive.zip!/plots/intensity/qualityPlots/peptides/GCSP_Cre12.g534800.t1.1.html]

### Cre12.g534800.t1.1

Trivial name: GCSP  
SeqCoverage [median]: 29.30  
Mass: 111.35 kDa  
  
 

| Sequence | MissedCleavage | Mass [Da] | wtIntensity | muIntensity | Score |
| --- | --- | --- | --- | --- | --- |
| AAFPAPWVR | 0 | 1013.54 | 3095.25 | 7098.97 | 76.03 |
| AEGLGLEAVVVDEDK | 0 | 1542.78 | 475.62 | 2153.37 | 177.10 |
| AEGLGLEAVVVDEDKMAYAK | 1 | 2107.05 | 0.00 | 12266.40 | 113.53 |
| AFHLSR | 0 | 729.39 | 698.85 | 0.00 | 91.58 |
| AHLAPFLPTHPVVPTGALPSR | 0 | 2177.21 | 0.00 | 8431.85 | 105.77 |
| ATGFGSLDALIDATVPK | 0 | 1674.88 | 1686.48 | 0.00 | 148.96 |
| DGDADKYVALALNEK | 1 | 1620.80 | 2181.39 | 15119.43 | 79.35 |
| DGMNLGK | 0 | 733.34 | 2779.32 | 1276.09 | 167.82 |
| EEIREIESGK | 1 | 1188.60 | 523.90 | 0.00 | 38.40 |
| EEIREIESGKADK | 2 | 1502.76 | 317.22 | 0.00 | 51.61 |
| ERAAFPAPWVR | 1 | 1298.69 | 0.00 | 5338.91 | 56.36 |
| ETAGIEAEDVAK | 0 | 1231.59 | 2991.29 | 5493.30 | 141.88 |
| ETAGIEAEDVAKR | 1 | 1387.69 | 1035.03 | 0.00 | 50.56 |
| HAPHAPGVVLADK | 0 | 1310.71 | 0.00 | 2560.87 | 84.61 |
| HAPHAPGVVLADKWERPYSR | 2 | 2285.18 | 0.00 | 1990.35 | 48.88 |
| HNSGTPAEIDSMVK | 0 | 1484.69 | 12978.08 | 13067.24 | 152.11 |
| IIGMSIDAQGKPALR | 1 | 1568.87 | 18205.47 | 29223.35 | 113.35 |
| IVTVSTDSQGNVNIPELR | 0 | 1941.02 | 1569.35 | 2558.21 | 121.04 |
| KSTFLQQPIFNTYHNEHDMLR | 1 | 2618.27 | 0.00 | 3152.21 | 49.09 |
| LGHTVPSAPFFDTVSVTVK | 0 | 2001.06 | 680.01 | 0.00 | 45.70 |
| STFLQQPIFNTYHNEHDMLR | 0 | 2490.18 | 0.00 | 4516.45 | 64.83 |
| SYIGMGYYGTHVPNVILR | 0 | 2039.03 | 0.00 | 25654.50 | 131.18 |
| VDNVYGDR | 0 | 936.43 | 13301.74 | 8479.52 | 177.97 |
| VDNVYGDRHLITR | 1 | 1556.81 | 0.00 | 4490.22 | 95.65 |
| VNGLASVFAAGAAK | 0 | 1274.70 | 806.64 | 7496.22 | 97.45 |
| WDNGTSAEAVAAHA | 0 | 1398.62 | 585.28 | 0.00 | 135.86 |
| YHEGMTESQFLEYFK | 0 | 1907.84 | 0.00 | 13158.70 | 126.71 |
|  |  |  | 63910.9 | 173526.2 |  |
